# Phase encoded quantum key distribution up to 380 km in standard telecom grade fiber enabled by baseline error optimization

**DOI:** 10.1038/s41598-023-42445-y

**Published:** 2023-09-22

**Authors:** Nishant Kumar Pathak, Sumit Chaudhary, Sangeeta, Bhaskar Kanseri

**Affiliations:** 1 Experimental Quantum Interferometry and Polarization (EQUIP), Department of Physics, Indian Institute of Technology Delhi, Hauz Khas, New Delhi, 110016 India; 2 Optics and Photonics Centre, Indian Institute of Technology Delhi, Hauz Khas, New Delhi, 110016 India

**Keywords:** Quantum information, Fibre optics and optical communications

## Abstract

Phase encoding in quantum key distribution (QKD) enables long-distance information-theoretic secure communication in optical fibers. We present a novel theoretical model characterizing errors from various sources in practical phase encoding-based QKD systems, namely the laser linewidth, detector dark counts, and channel dispersion. This model provides optimized optical pulse parameters and less distortion in pulses, which eliminates system imperfections and leads to a reduced quantum bit error rate (QBER) for practical QKD scenario. This analysis is applicable to various fiber-based phase and time encoding protocols. In particular, we implement this to a differential phase shift (DPS) QKD scheme operating at a 2.5 GHz clock, which produces a secure key rate of 193 bits/s at a fiber length of 265 km and an unprecedented QBER < 1$\%$ up to 225 km length with standard telecom components. We show that by adjusting the quantum efficiency and dark count rates of detectors, proposed system can establish secure keys up to 380 km distance using standard telecom grade fiber with a QBER of 1.48%. Moreover, the system is compatible with existing optical fiber networks and capable of establishing a secure key exchange between two cities 432 km apart using ultra-low-loss (ULL) specialty fiber.

## Introduction

Quantum Key Distribution (QKD) is a cryptographic method based on the principles of quantum mechanics^1, 2^ that enables two parties to securely share a secret key for encrypting and decrypting messages^3^. Any attempt of eavesdropping, therefore, introduces detectable error into the system, alerting legitimate users. Qubits can be encoded in various degrees of freedom, including polarization, phase, time bin, etc., and distributed through fiber or free space quantum channels. Fiber-based QKD serves as a perfect candidate for inter-city quantum communication. In single-mode fiber, stress induced from the environment randomly evolves the birefringence causing the polarization drift. Therefore, additional resources are required for polarization compensation in polarization encoding based QKD. In contrast, the phase encoding based QKD system is less susceptible to noise and other types of interferences. It is diminutively affected by polarization drift, making it a perfect fit for fiber-based channels. Various protocols exist which utilize phase encodings such as phase-based BB84^4^, DPS and several variants of DPS protocol^5^. There have been many recent advancements in QKD, such as investigating QKD security^6^ with realistic devices^7^, QKD using random states^8^, analysis of reference frame independent QKD^9, 10^ and decoy-state method for quantum private query^11^. Significant progress has been achieved in realizing long range twin field QKD without quantum repeaters^12^, and MDI QKD with polarization-discriminated time-bin phase encoding^13^. The transmission of polarization states from GEO satellite to earth has been demonstrated^14^, and chip-based QKD platform has been studied^15^ that is compatible with current telecommunication hardware for hybrid classical and quantum communication.

One of the most promising implementations of phase-encoded QKD is differential phase shift QKD (DPS-QKD), known for its implementable simplicity and high key rate^16^. It is also resistant to various attacks, including those based on photon-number-splitting (PNS)^17^ and general individual attacks^18^. DPS-QKD is based on the phase difference between adjacent pulses of a coherent pulse train having applications in various domains, including secure communication, network security, and quantum cryptography. In secure communication, DPS-QKD can be used to establish secure links between two parties, protecting sensitive information such as financial transactions, medical records, and government secrets. DPS-QKD can also secure network communication, such as in the case of secure routing and authentication in the internet of things (IoT) and other communication networks^19^. It has several use cases with various cryptographic protocols, such as quantum digital signatures^20–22^, and quantum secret sharing^23, 24^.

Recent research has focused on enhancing the performance and security of DPS-QKD by several modifications in the original DPS protocol^5^. For instance, round-robin DPS (RRDPS), first introduced by Sasaki et al.^25^, has been widely explored. Although it offers a robust security aspect, the implementation is resource intensive. The typical realizations are having nearly 100 km channel lengths or are limited in secure key rates^26–31^. Not to mention, the Tokyo field demonstration^32^ implementing DPS protocol was also limited to 90 km. Such shorter channel lengths in the above experiments require several trusted nodes to be implemented to realize long-distance quantum communication making the system prone to compromise in security. Finite secure key rate generation was reported with a clock rate of 1 GHz^33^, which used special dispersion shifted fibers (DSF) instead of standard telecom fibers. Using specialty fibers such as DSF or ultra-low loss (ULL) fibers would require a new telecom infrastructure with huge monetary requirements. The real test of a QKD implementation would be with standard telecom fibers, as quantum channels are already available in the existing telecom infrastructure which support electronics with a small footprint. Moreover, the best performance of DPS QKD utilizing regular telecommunications fiber has been so far limited to a quantum bit error rate (QBER) of $3.45\%$ and a maximum channel loss of 52.9 dB^28^.

The noise in a realistic QKD setup arises from imperfections in devices. Earlier studies in DPS QKD^34^ only assume a baseline noise level and error from the detector dark counts. This simple theoretical model is widely used in fitting experimental data, but it lacks the ability to analyze the whole experimental setup and optimize it. The above implementations of the DPS-QKD protocol have been done with the same old theory, which does not consider the various sources of errors. Such limitations exist for a wide range of QKD protocols that use components like modulators and interferometers, for example, phase-encoded BB84, coherent one-way (COW), RRDPS, MDI QKD, quantum private query (QPK) protocols, etc^11, 35–38^. Thus a thorough study of all major sources of errors is essential for the efficient implementation of such QKD protocols for long distances.

In this work, we aim to analyze device imperfections and characterize bit errors caused by source linewidth, electro-optical modulation, chromatic dispersion, and detector’s dark counts to optimize performance for high secure key rates and low QBER in a phase-based QKD system. This analysis allows us to precisely characterize phase-encoded QKD systems taking the DPS QKD system as an example. With this optimization, QBER of $2.36\%$ is achieved at 265 km fiber channel, which is at least 31$\%$ improvement over previously reported works^28^. This system can generate a secure key rate of 192.7 bits/s, which has not been achieved yet for such lengths as per the authors’ knowledge. By lowering the bias current of the superconducting nanowire single photon detectors (SNSPDs), the QKD setup enables secure key distribution for channel loss of more than 72.2 dB, equivalent to a channel length of 380 km with QBER nearly 1.48$\%$. Such capacity will also enable DPS QKD based long-distance quantum secret sharing^24, 39^ and quantum digital signature^22, 40^, including other useful applications like quantum secure direct communication and quantum conference key agreement. With a similar setup as this experiment except for the fiber channel, this analysis will enhance the performance for free space DPS-QKD enabling high key rate GEO satellite to ground secure key distribution^41^.

This paper is organized as follows: “Theoretical model” starts with an introduction to DPS QKD, and then presents a detailed analysis of QBER calculations incorporating device imperfections. Here, the QBER is characterized in three different categories. In “Experiment and results”, the experimental setup is highlighted and the achieved results are discussed. The comparison of our results with those reported in literature is made in “Discussion”. Finally, “Conclusion” summarises the overall findings.

## Theoretical model

The performance of QKD systems has extraordinarily improved over time in terms of length and key rate due to technological advancements. QBER is the limiting factor that bounds the QKD performance, which originates in the system due to imperfections in real-time experimental devices. In order to optimize a QKD protocol, it is essential to study the origin of QBER due to various components. In the simple conventional approach^28–31, 34, 42^, QBER constitutes system baseline error and dark counts of detectors. This baseline error is constant for the system, but the effect of dark counts becomes dominant as distance increases. To get a deep understanding, the QBER arising from various imperfections in the devices is rigorously calculated, making the analysis more precise and realistic for practical scenarios. This analysis aims to optimize the baseline error focusing on DPS QKD, which can also be applied to various other protocols using similar components.

### DPS QKD protocol

**Figure 1:**
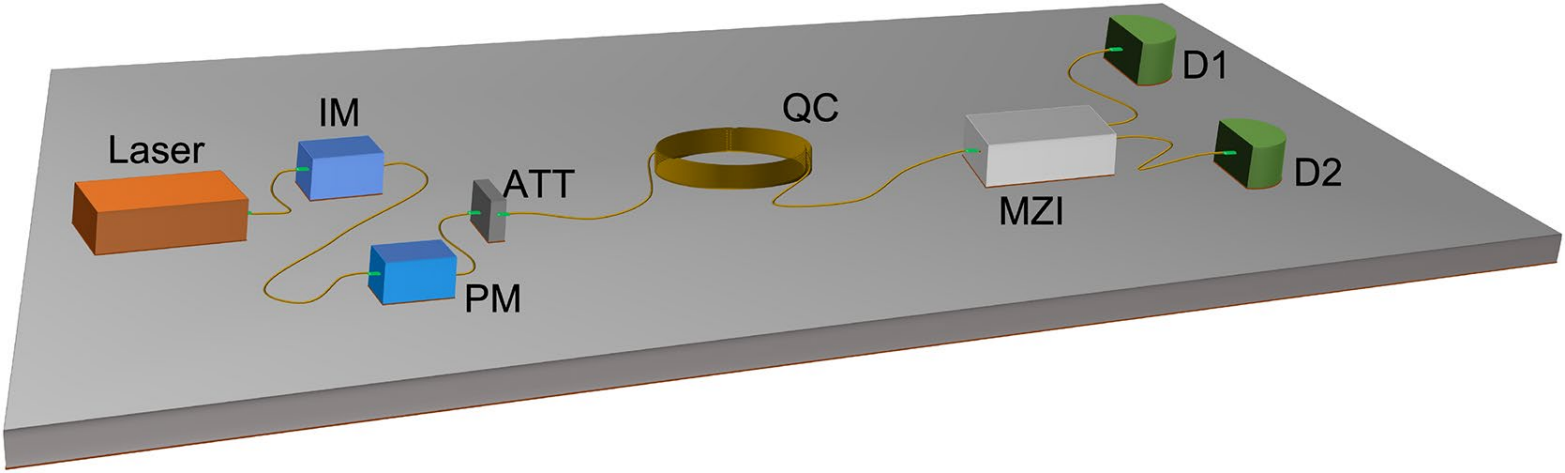
Basic configuration of a DPS QKD protocol. D1 clicks for 0 phase difference, and D2 clicks for $\pi$ phase difference between adjacent pulses. *IM* Intensity modulator, *PM* phase modulator, *ATT* optical attenuator, *QC* quantum channel, *MZI* Mach Zehnder Interferometer, *D1, D2* single photon detectors.

The basic scheme of the DPS protocol is shown in Fig. 1. A continuous wave laser is modulated into a train of pulses with an intensity modulator (IM). The pulses further pass through a phase modulator where a random phase 0 or $\pi$ is given to each pulse. The pulses are then attenuated to a single photon regime depending on the security requirements, usually with nearly 0.2 mean photons. The encoded states become of the form $\vert \alpha e^{i\phi _j}\rangle _{0,j}\vert \alpha e^{i\phi _{j+1}}\rangle _{0,j+1}$ where $\phi \in \{0, \pi \}$ denotes the phase of *j*th pulse, and $\alpha$ denotes the amplitude of the weak coherent pulse. The encoded weak coherent state propagates through the fiber quantum channel, which then enters Bob’s one-bit-delay Mach Zehnder Interferometer (MZI). Inside the one-bit delay MZI, each pulse splits at the first beam splitter and interferes with its neighboring (next) pulse at the second beam splitter. The post-selected state thus becomes $\vert \frac{\alpha }{2}(e^{i\phi _j}+e^{i\phi _{j+1}})\rangle _{D1,j+1} \vert \frac{i\alpha }{2}(e^{i\phi _j}-e^{i\phi _{j+1}})\rangle _{D2,j+1}$. According to the phase difference between adjacent pulses being 0 or $\pi$, the photon is detected in either of the detectors D1 or D2. Bob shares his detection timestamps with Alice (but no information about which detector has clicked). Alice discards the bits that are not detected by Bob. This way, both arrive at a common sifted key sequence with some error given by QBER.

In this analysis, the key rate and QBER for DPS QKD are calculated, incorporating the effect of the properties of the light source, electro-optic intensity and phase modulation, dispersion in optical fiber channel, Mach Zehnder interferometer, and the single photon detectors. QBER is categorized into three categories $QBER_{disp}$, $QBER_{MZI}$, and $QBER_{dark}$ to finally calculate the total QBER of the system. $QBER_{disp}$ incorporates the effect of imperfection in phase modulation, optical pulse shape, and dispersion in fiber channels. $QBER_{MZI}$ comprises the effect of source linewidth on the Mach-Zehnder interferometer, and $QBER_{dark}$ contains the effect of dark counts in the single photon detector. The key rate depends on the losses in the system, the dead time of detectors $t_{deadtime}$, and the width of the detection window $t_W$. The theoretical proof for unconditional security of DPS QKD is still being explored with various assumptions^6^. However, for the scope of this work, we have considered the theoretical analysis given in Ref.^18^, which provides the security guarantee under general individual attacks. The required mean photon number is calculated for secure key distribution under these attacks. Experimentally fixing the mean photon number using a set of optical attenuators suffices the security under these attacks.

### Effect of dispersion and electro-optic modulation

The performance of the fiber optic communication system is limited by the pulse broadening induced by the dispersion effect. At telecom wavelength in standard fibers, pulse broadening is substantially caused by second-order dispersion. This broadening causes a portion of adjacent pulses to intermix, thus limiting transmission length for a particular bit rate. Apart from this, polarization mode dispersion (PMD) is induced by random polarization evolution in fibers manifested by material anisotropy or asymmetric stress that distorts the pulse shape. However, PMD effects are inconsiderable in standard telecom fibers compared to chromatic dispersion.

**Figure 2:**
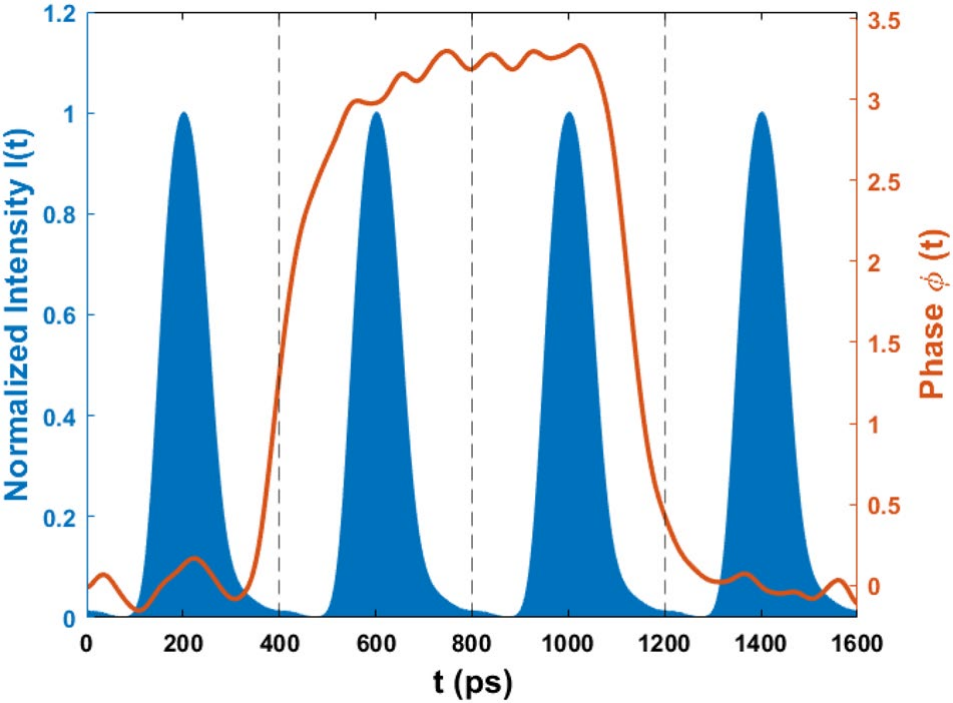
Intensity and phase modulation. Optical pulses (blue area) are produced from the intensity modulation at 2.5 GHz. The intensity profile is denoted by the function I(t). Phase modulation of optical pulses (red curve) is represented by $\varphi (t)$.

**Figure 3:**
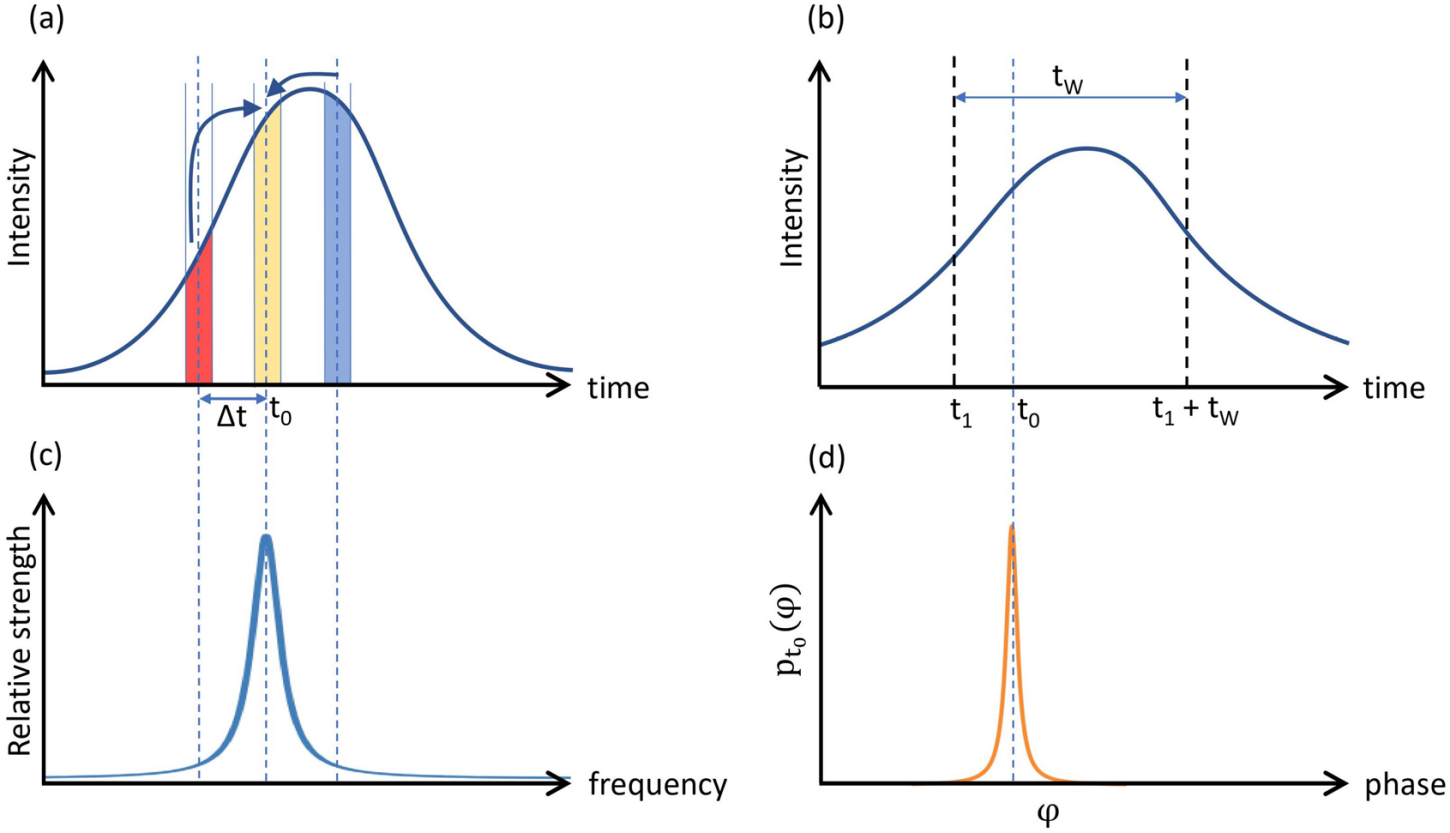
The effect of dispersion and practical phase modulation. (**a**) The curve represents the temporal pulse shape before dispersion. After dispersion in the medium at time instant $t_0$ pulse will also have photons from nearby regions ($t_0 - \Delta T$) as higher wavelength photons (red color) travel faster and lower wavelength photons (blue color) travel slower. (**b**) The temporal shape of the dispersed pulse (broadened), and $t_W$ is the detection window size. (**c**) The spectrum of pulses governs the dispersion. (**d**) As a result of dispersion at time $t_0$, the pulse will have a phase distribution as $p_{t_0 }(\varphi )$.

Intensity or phase modulation of CW laser to produce optical pulses also modulates the spectrum of light that is mainly governed by the modulation depth and modulation frequency. The output spectrum consists of sidebands separated by the modulation frequency and their relative power depends on the modulation depth^43^. Ideally, the phase modulation should be a random step function (0 or $\pi$) but in actual implementation, electronic signals are constrained by significant fall and rise times. Thus, adjacent coherent optical pulses don’t exhibit purely 0 or $\pi$ phase difference and contribute to QBER. Electro-optical modulation broadens the spectral width of optical pulses that will cause dispersion in the fiber channel. This QBER contribution is considered as $QBER_{disp}$ and calculated as following:

Suppose I(t) is the intensity of the optical pulse and $\phi (t)$ is the phase given to the optical pulse at time t as shown in Fig. 2. $\phi (t)$ is obtained by measuring the waveform signal input to the phase modulator and multiplying the proportionality constant estimated while calibrating the phase modulator. $S^\prime (\nu )$ is the spectral profile of photons after the intensity and phase modulation as shown in Fig. 3c. T is the time period of the optical pulse train.

In a one-bit delay MZI, two optical pulses separated by time T will interfere. Since the phase difference between two adjacent pulses is not precisely 0 or $\pi$, photons have a finite probability of going into the wrong detector. When considering the effect of dispersion, photons of different wavelength travel at different speeds, and the temporal broadening of the pulse is observed. Let us assume that the initial pulse entering into the fiber channel (shown in Fig. 3a) has phase $\phi (t_0)$ at a time $t_0$. While propagating into the optical fiber channel, photons of various wavelengths will travel at different speeds causing the broadening of the pulse as shown in Fig. 3b. In the broadened pulse, at time label $t_0$, there would be a fraction of photons that traveled at different speeds in the medium. So, the broadened pulse at time label $t_0$, contains a fraction of photons from the time label $t_0+\Delta t$ of the initial pulse as shown in Fig. 3a and that component is written as:

1 $$\begin{aligned} {p(t_0,\ \mathrm {\Delta t})\ =\ I(t_0+\mathrm {\Delta t})S^\prime ({\lambda }_c + \frac{\mathrm {\Delta t}}{\alpha _{disp} L}), } \end{aligned}$$

where, $\alpha _{disp}$ is the dispersion coefficient, L is fiber length, $\lambda _c$ is the central wavelength. The broadened pulse at time label $t_0$ namely $p(t_0, \Delta t)$ contains the fraction of photons that have a phase $\phi (t_0+ \Delta t)$ of the initial pulse. Thus by varying $\Delta t$ for the whole range, phase distribution $p_{t_0}(\varphi )$ at time label $t_0$ of the pulse after dispersion is obtained as depicted in Fig. 3d. It is worth noting that $\phi (t)$ is a function of phase imparted by phase modulator and should not be confused with variable $\varphi$ in phase distribution function $p_{t_0}(\varphi )$. This shows that the initial modulated pulse has a fixed phase value at any point in time, whereas in the broadened pulse, there is a distribution of phase as a consequence of group velocity dispersion. In a similar manner, the phase distribution $p_{t_0+T}(\varphi )$ can be obtained for time $t_0 + T$ that corresponds to photons in the adjacent pulse. The interference at the second beam splitter of MZI is the interference of these two phase distributions $p_{t_0}(\varphi )$ and $p_{t_0+T}(\varphi )$. The phase difference between two adjacent pulses is the convolution of $p_{t_0}(\varphi )$ and $p_{t_0+T}(\varphi )$.

2 $$\begin{aligned} p_{diff,t_0}(\varphi )\ =\ \ \int _{-\infty }^{\infty }{p_{t_0}(\varphi )p_{t_0+T}(\xi \ -\ \varphi )d\xi }. \end{aligned}$$

For the interference between two adjacent pulses (say *i*th pulse and $i^{th}+1$ pulse), let $N^{(i)}_1$ and $N^{(i)}_2$ are photons reaching D1 and D2 respectively for the time detection window $t_W$ given by

3 $$\begin{aligned} N_1^{(i)} = \int _{t_1}^{t_1+t_W}{\int _0^{2\pi }{p_{diff,{\ t}_0}(\varphi ){cos}^2(\frac{\varphi }{2})}d\varphi \ dt_0}, \end{aligned}$$

4 $$\begin{aligned} N_2^{(i)} = \int _{t_1}^{t_1+t_W}{\int _0^{2\pi }{p_{diff,{\ t}_0}(\varphi ){sin}^2(\frac{\varphi }{2})}d\varphi \ dt_0}. \end{aligned}$$

The amount of photons leaking to the wrong port of MZI due to phase imperfection is given by $min(N^{(i)}_1, N^{(i)}_2)$. Here, *min*( ) function is used since we assume a small fraction of photons is leaking into the wrong port of detection due to phase imperfection and channel dispersion and the majority of the photons follow the phase encoding. The corresponding *QBER* is given by

5 $$\begin{aligned} QBER_{disp}^{(i)} = \frac{min(N_1^{(i)},N_2^{(i)})}{N_1^{(i)}+N_2^{(i)}}. \end{aligned}$$

By considering the interference between adjacent pulses for a large train of optical pulses, fluctuations are averaged out in the estimation of $QBER_{disp}$. Taking the ensemble average of a long train of optical pulses yields

6 $$\begin{aligned} QBER_{disp} = \langle \frac{min(N_1^{(i)},N_2^{(i)})}{N_1^{(i)}+N_2^{(i)}} \rangle _i. \end{aligned}$$

**Figure 4:**
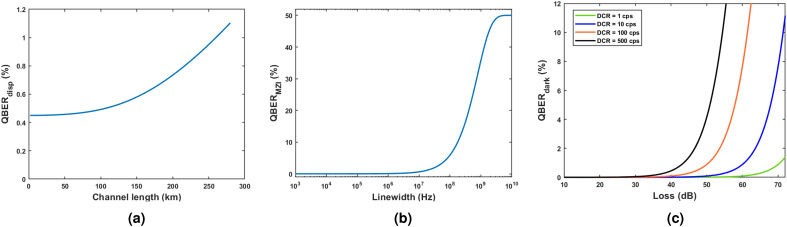
Characterization of QBER into three categories. (**a**) Effect of $QBER_{disp}$ with channel length. At 0 km length, $QBER_{disp}$ is only due to imperfect phase modulation, and with distance, it increases as dispersion occurs along the channel. This curve is plotted for an 18 ps/km-nm dispersion coefficient and a Gaussian spectral width of 0.0163 nm (FWHM). (**b**) Variation of $QBER_{MZI}$ as a function source linewidth for a free spectral range of 2.5 GHz. (**c**) $QBER_{dark}$ as a function of total losses in the QKD system.

For phase difference of 0 (or $\pi$) between adjacent pulses, photons going to the wrong detector D1 (or D2) will contribute in ${QBER}_{disp}$. Also, the area under the curve from $t_1$ to $t_1+t_W$ of the broadened pulse is defined as *f* as shown in Fig. 3b, and it is the fraction of photons considered for key generation, and other photons are discarded since they belong to the time regions of comparatively large QBER. In Fig. 4a, one can see the variation of $QBER_{disp}$ with the channel length. To mitigate the dispersion effects, a dispersion compensating fiber (DCF) could be added to the scheme.

### Effect of source linewidth on Mach Zehnder interferometer

The linewidth of the laser source originates from the phase noise inside the laser^44^, which impacts the performance of MZI. The transmittance of MZI can be expressed in terms of losses in MZI arms, their extinction ratio, and free spectral range ($\nu _{fsr}$). The higher linewidth of the source causes photons to leak in the wrong output port of MZI and is denoted by $QBER_{MZI}$. The interferometer has two arms, out of which one is delayed with respect to the other by one pulse separation. Let the two arms of MZI have insertion losses $IL_1$ and $IL_2$, and extinction ratios $ER_1$ and $ER_2$. Thus the transmittance ($T_1$ and $T_2$) of the output ports of MZI can be expressed as:

7 $$\begin{aligned} T_1(\nu )\ =\ \ {10}^\frac{({IL}_1-\ {ER}_1)}{10}\ +\ \ {10}^\frac{{IL}_1}{10}[1\ -\ {10}^\frac{-{ER}_1}{10}\ ]sin^2(\frac{\pi \nu }{\nu _{fsr}}), \end{aligned}$$

8 $$\begin{aligned} T_2(\nu )\ =\ \ {10}^\frac{({IL}_2-\ {ER}_2)}{10}\ +\ \ {10}^\frac{{IL}_2}{10}[1\ -\ {10}^\frac{-{ER}_2}{10}\ ]cos^2(\frac{\pi \nu }{\nu _{fsr}}). \end{aligned}$$

Since the source has some finite spectral width, photons will leak into the wrong output port of the Mach-Zehnder interferometer. Let us define four coefficients $m_{ij},\ \text{where}\ i,j\ \in \{1,\ 2\}$ representing the fraction of photons supposed to go in output port *i* actually going to output port *j* as follows:

9 $$\begin{aligned} m_{11}\ =\ \int _{-\infty }^{\infty }{S(\nu \ -\ \frac{\nu _{fsr}}{2})T_1(\nu )d\nu }, \end{aligned}$$

10 $$\begin{aligned} m_{12}\ =\ \int _{-\infty }^{\infty }{S(\nu )T_1(\nu )d\nu }, \end{aligned}$$

11 $$\begin{aligned} m_{21}\ =\ \int _{-\infty }^{\infty }{S(\nu \ -\ \frac{\nu _{fsr}}{2})T_2(\nu )d\nu }, \end{aligned}$$

12 $$\begin{aligned} m_{22}\ =\ \int _{-\infty }^{\infty }{S(\nu )T_2(\nu )d\nu }, \end{aligned}$$

where, $S(\nu )$ is the spectral width of the laser. The fraction of photons that have leaked into the wrong output port of MZI, which contributes to QBER, can be expressed as

13 $$\begin{aligned} {QBER}_{MZI}\ =\ \frac{m_{12}\ +\ m_{21}}{m_{11}+\ m_{12}+m_{21}+m_{22}}. \end{aligned}$$

In Fig. 4b, the behavior of $QBER_{MZI}$ with source linewidth is plotted for the MZI used in the experiment. The plot shows that the contribution to $QBER_{MZI}$ is minimally affected by an increase in linewidth for spectral widths much smaller than the free spectral range of the MZI. As the linewidth increases further, the QBER linearly increases and saturates at 50$\%$.

### Effect of detector dark counts

The dark counts generated in a single photon detector are random false detections that do not correspond to a signal photon. These dark counts give rise to QBER that is calculated below:

Suppose $\eta _{detector}$ is the quantum efficiency, $t_{deadtime}$ is the dead time, and r$_{DCR}$ is the dark count rate of the single photon detector. Let $r_{thermal}$ be the rate of thermal photons generated inside a single photon detector that triggers an avalanche. The total photon rate that could cause an avalanche in a single-photon detector is denoted by $\mathrm {\Lambda }$ that majorly consists of signal photons,

14 $$\begin{aligned} \Lambda = \dfrac{1}{2} [(1 - e^{-\mu \eta _{link} \eta _{detector}})R + 2 r_{thermal}], \end{aligned}$$

where the 1/2 factor is due to the equiprobable distribution of incoming photons in both detectors due to random phase encoding. $\eta _{link}$ is the channel efficiency considering the fiber loss and any additional losses in the system and *R* is the clock speed. The total photon count rate registered by the detection system is defined as the sifted key rate given by:

15 $$\begin{aligned} R_{sifted}=2f\alpha _{deadtime} \Lambda = f \alpha _{deadtime}[(1 - e^{-\mu \eta _{link} \eta _{detector}})R + 2 r_{thermal}]. \end{aligned}$$

Here, $\alpha _{deadtime} = e^{-\Lambda t_{deadtime}}$^45^, and $r_{thermal}$ can be calculated from the Eq. (15) by substituting $\eta _{link} = 0$ and $R_{sifted} = 2r_{DCR}$. *f* is defined in “Effect of dispersion and electro-optic modulation” as the fraction of photons inside the detection window considered for key generation. QBER due to dark counts of single photon detectors is given by:

16 $$\begin{aligned} QBER_{dark} = \dfrac{r_{thermal}}{2 \Lambda }. \end{aligned}$$

The variation of $QBER_{dark}$ with system losses are presented in Fig. 4c for various detectors’ dark count rates. It shows that $QBER_{dark}$ becomes significant when the rate of signal photons is comparable to the dark count rate at higher channel losses.

### Estimation of QBER and key rate

Recalling the definition, QBER is the fraction of detected photons that are inconsistent with Alice’s encoding. Half of the detected dark counts contribute to QBER denoted as $QBER_{dark}$, and the probability that a detection occurred due to signal photon is $(1-2QBER_{dark})$ .

Let us consider $QBER_{dark}$, $QBER_{disp}$ and $QBER_{MZI}$ to be independent events. A signal photon going into the wrong port due to $QBER_{disp}$ has a probability of $(1-QBER_{MZI})$ to pass unaffected. The net QBER becomes $QBER_{disp} (1-QBER_{MZI})$. Similarly, a signal photon correctly passing due to dispersion with probability $(1-QBER_{disp})$ has a probability $QBER_{MZI}$ of flipping to the wrong port. The net QBER, in this case becomes $(1-QBER_{disp})QBER_{MZI}$. Adding up all the contributions, collectively total QBER of the system can be written as:

17 $$\begin{aligned} \begin{aligned} QBER =&QBER_{dark} +(1-2QBER_{dark})(QBER_{MZI} + QBER_{disp} - 2QBER_{MZI}QBER_{disp}). \end{aligned} \end{aligned}$$

The factor $(1-2QBER_{dark})$ is the probability that a detection occurred due to a signal photon. Figure 4 shows the characteristics of all three sources of QBER discussed above. The secure key rate can be calculated as^18^:

18 $$\begin{aligned} R_{secure} = R_{sifted}(\tau (QBER) {-} f_{EC}H(QBER)), \end{aligned}$$

where $R_{sifted}$ is calculated in Eq. (15), $\tau$ is the compression factor for privacy amplification^46^, $f_{EC}$ is the factor due to the efficiency of the error correction process^47^ and is chosen to be 1.16 in this analysis, considering the performance of the bidirectional error reconciliation protocol^48^ . H(x) is the binary entropy function. A better estimate of the factor $f_{EC}$ can be made taking into account the finite block size effect and QBER of the protocol^49^. Since the analysis explores only the sources of QBER, it is compatible with established methods of determining phase error rates and later advancements in security analysis through Eq. (18).

**Figure 5:**
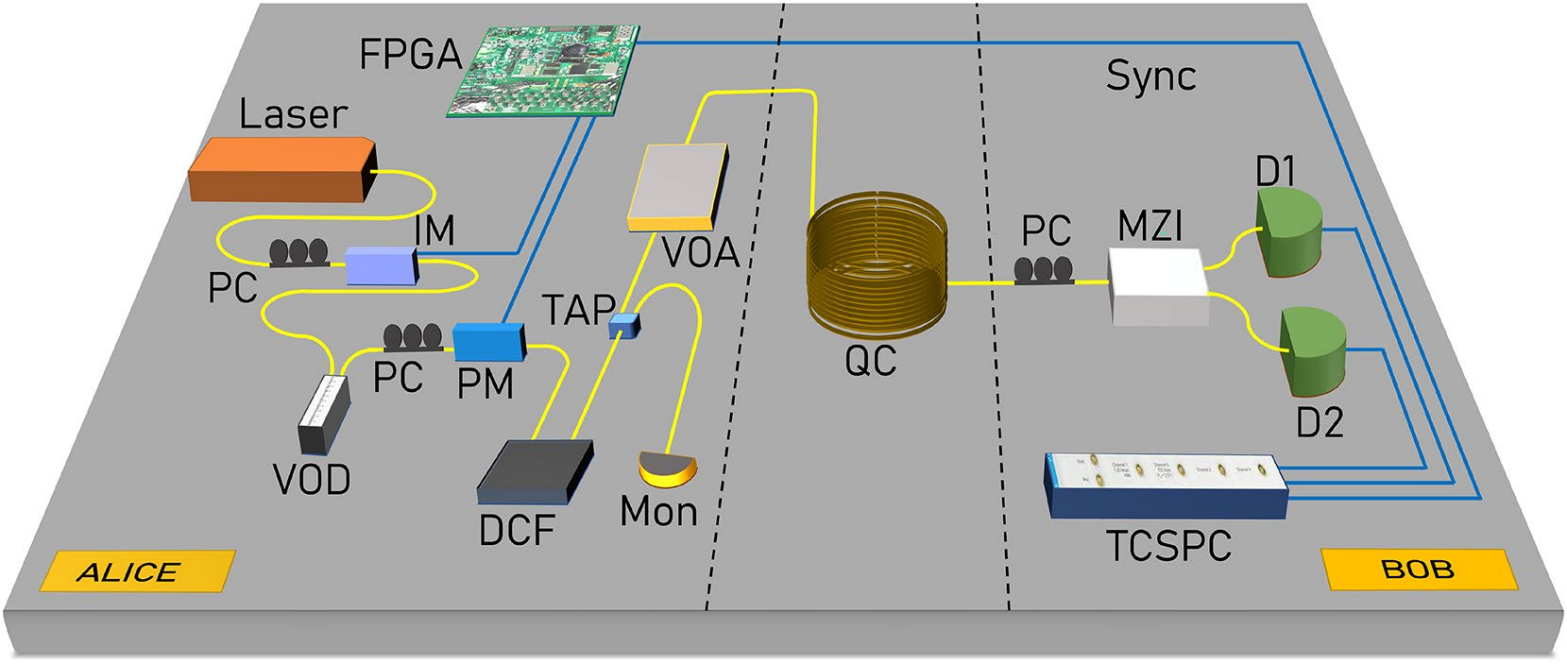
2.5 GHz clock DPS-QKD experimental setup synchronized by FPGA and using telecom grade fiber as a quantum channel. *PC* polarization controller, *Mon* power monitor, *IM* intensity modulator, *PM* phase modulator, *VOD* variable optical delay line, *VOA* variable optical attenuator, *TAP* 90:10 beam splitter, *MZI* Mach-Zehnder interferometer, *D1, D2* superconducting nanowire single-photon detectors, *TCSPC* time-correlated single photon counting, *FPGA* field programmable gate array, *Sync* synchronization & classical channel, *QC* fiber quantum channel.

## Experiment and results

A CW laser centered at ITU channel 22 was first modulated into a 2.5 GHz, 120 ps pulse train by an intensity modulator (IM). The IM was driven by a field programmable gate array (FPGA) as shown in Fig. 5. A phase modulator (PM) encoded the pulses with random bit sequences with a phase of either 0 or $\pi$. The encoded pulse train is then attenuated by a variable optical attenuator (VOA) that sets the required mean photon number using a power meter. The mean photon per pulse was set to 0.23 for fiber lengths up to 175 km and 0.24 beyond that length as calculated from the theory considering general individual attacks^18^. A dispersion compensation fiber (DCF) up to 120 km length was used before the variable optical attenuator (VOA) to compensate for chromatic dispersion at larger lengths. Placing the DCF before quantum channel is important to avoid introducing extra losses in the channel.

At Bob’s end, a 1-bit delay MZI is used so that even and odd pulses interfered with one another. The 1-bit delay MZI used in this experiment is a commercially available off-the-shelf fiber coupled MZI (make Kylia). The interferometer is thermally stabilized to avoid any phase drift. The phase of the MZI is tuned using a resistive heater placed in one of its arms. The delay between the arms corresponds to the temporal separation of pulses generated by the intensity modulator (IM) at 2.5 GHz. The two outputs of the MZI are then connected with SNSPDs separately. Polarization controllers were placed before the intensity modulator, phase modulator, and MZI to diminish the polarization effects in these devices. The time stamps of clicks were recorded and analyzed by Bob using an FPGA-based time-correlated single photon counting module (TCSPC). Bob’s TCSPC is externally synchronized by Alice’s FPGA, which is also driving the intensity modulator and phase modulator.

**Figure 6:**
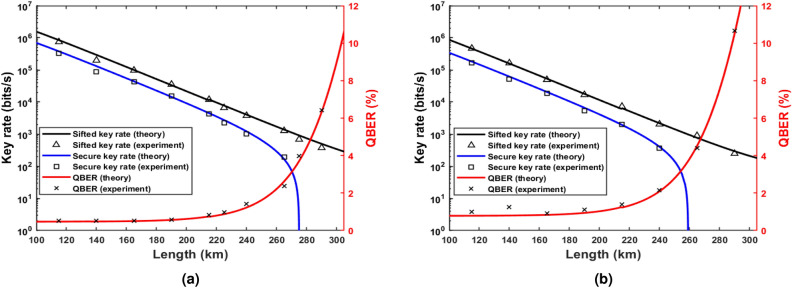
Performance of DPS QKD with two different laser sources with (**a**) 10 kHz linewidth and (**b**) 4 MHz linewidth. Theoretically predicted sifted key rate (black), secure key rate (blue), and QBER (red) variation with the losses in the system. Experimentally obtained sifted key rate (triangle), secure key rate (square), and QBER (cross) variation with the losses in the system are shown in the plots.

During the experiment, Alice and Bob generated keys and measured the QBER and sifted key rates by directly comparing their keys over fiber lengths ranging from 115 km to 290 km. A QBER of $2.36\%$, a sifted key rate of 1.38 kbits/s, and a secure key rate of 193 bits/s were achieved for 265 km of telecom grade fiber for a laser of 10 kHz linewidth with the best optimization of all the components, see Fig. 6a. In Fig. 6a,b, the theoretical curves are plotted using Eqs. (15), (17), and (18). We also performed the experiment with a 4 MHz linewidth laser to investigate the effect of laser linewidth in DPS QKD. Equation (13) predicts $QBER_{MZI}$ for 10 kHz and 4 MHz linewidth lasers as 0.02$\%$ and 0.27$\%$, respectively. The QBER curves in Fig. 6a,b are validated by experimental data. An upward shift in QBER in Fig. 6b is thus an increment in the baseline error due to the effect of laser linewidth.

With a 4 MHz linewidth laser, a QBER of $3.25\%$, a sifted key rate of 1.16 kbits/s, and a secure key rate of 56.68 bits/s was achieved when the channel length was 255 km as shown in Fig. 6b. Using a DCF also enhances performance by controlling the pulse broadening. $QBER_{disp}$ curve in Fig. 4a quantitatively presents the QBER contribution from dispersion effect. At a channel length of 265 km, $QBER_{disp}$ is 1.03$\%$ without using DCF; when a DCF of length 120 km was used, the $QBER_{disp}$ component was reduced to 0.57$\%$.

**Figure 7:**
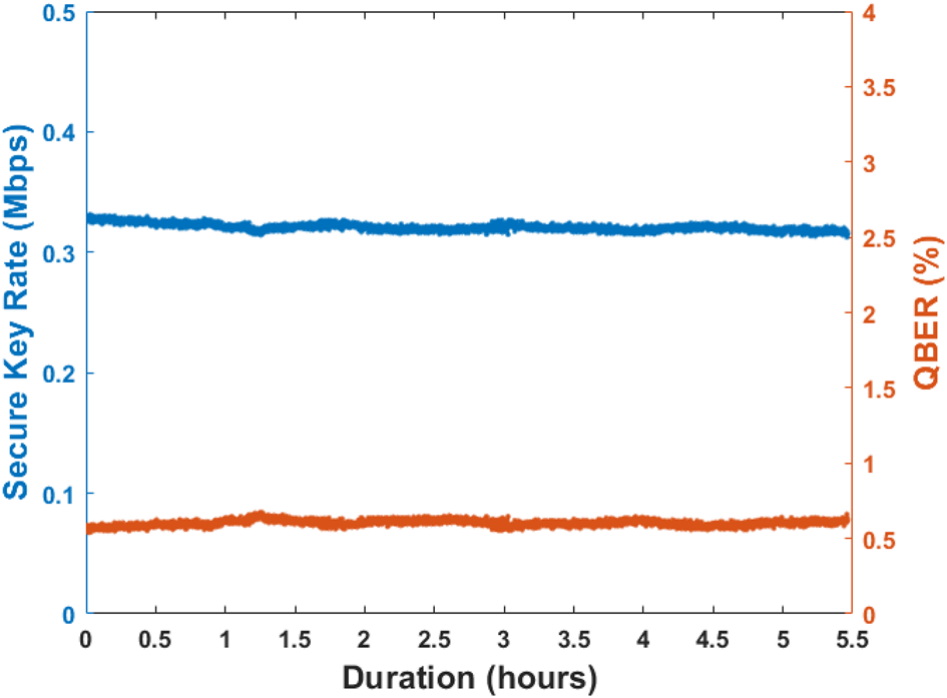
Variation of secure key rate and QBER measured for a duration of nearly 5.5 h showing a reasonably stable QKD system.

The stability of the setup was also observed in terms of QBER and secure key rate for a channel length of 115 km. The system was stable for more than 5 h, which proves the applicability of the DPS QKD system for long-hour operations. The standard deviation for fluctuations in QBER and key rates were 0.02$\%$ and 0.003 Mbits/s, respectively, as shown in Fig. 7. The high stability and low QBER allow the setup to withstand more environmental fluctuations making it suitable for long time secure key distribution.

### Measurement of spectral width after electro-optical modulation

**Figure 8:**
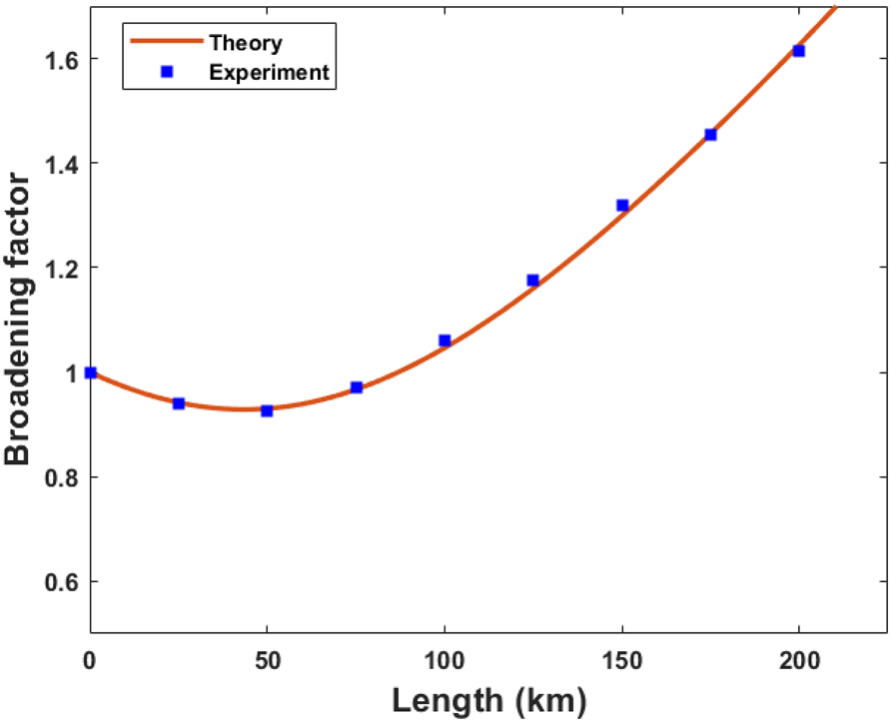
Broadening factor variation with the channel length in DPS QKD setup without using dispersion compensating fiber. Experimental data (blue square) is fitted with Eq. (19) represented by a solid curve (red) as explained in “Measurement of spectral width after electro-optical modulation”.

As mentioned in “Effect of dispersion and electro-optic modulation”, after intensity modulation and phase modulation, the inherent spectral width of the weak coherent pulses modifies. Intensity modulation generates sidebands in the spectrum at a separation of the frequency of the RF signal driving the intensity modulator. Irrespective of the inherent spectral width of the source, these modulation processes manipulate the spectrum. This new spectrum governs the effect of dispersion in the fiber channel. The resultant spectral width increases with the clock speed of the QKD protocol. Thus dispersion control is necessary for a high clock speed DPS QKD system. The broadening factor measures pulse width at any distance with respect to its initial width. In the same experimental setup in Fig. 5, we significantly increased the mean photon number and detected the photons using SNSPD at the output port of MZI. The pulse shape is constructed by analyzing the timestamp data and the pulse width is measured at several fiber lengths. The measured broadening factor is shown in Fig. 8. The propagation of optical pulses inside the single-mode fiber is described by the nonlinear Schrödinger equation. Since the working wavelength was far away from the zero dispersion point, the third-order dispersion effect could be neglected. The broadening factor is given by^50^

19 $$\begin{aligned} \frac{\sigma }{\sigma _0}\ =\ \sqrt{{(1\ +\ \frac{C\beta _2z}{\sigma _0})}^2\ +\ (1\ +\ {V_\omega }^2){(\frac{\beta _2z}{{2\sigma }_0})}^2}, \end{aligned}$$

where $\sigma _0$ and $\sigma$ are the pulse widths at distances 0 and *z* respectively, and C is the chirp parameter. $\beta _2 = {(\frac{d^2\beta }{d\omega ^2})}_{\omega \ =\ \omega _0}$ where $\beta$ is the mode propagation constant. $V_\omega \ =\ 2\sigma _0\sigma _\omega$, $\sigma _\omega$ is the spectral width. We used Eq. (19) to determine the spectral width of the optical pulse after modulation. From this measurement, $\sigma _\omega$ of 5.5 GHz was obtained and used in Eq. (1) to calculate $QBER_{pulse}$ which fits reasonably well with experimental results presented in Fig. 6. However, it is worth noting that MZI is not affected by this bandwidth since the sidebands in the spectrum are generated at a separation of the free spectral range of MZI. Therefore, only the linewidth of the laser has an effect on MZI as discussed in “Effect of source linewidth on Mach Zehnder interferometer”.

## Discussion

**Figure 9:**
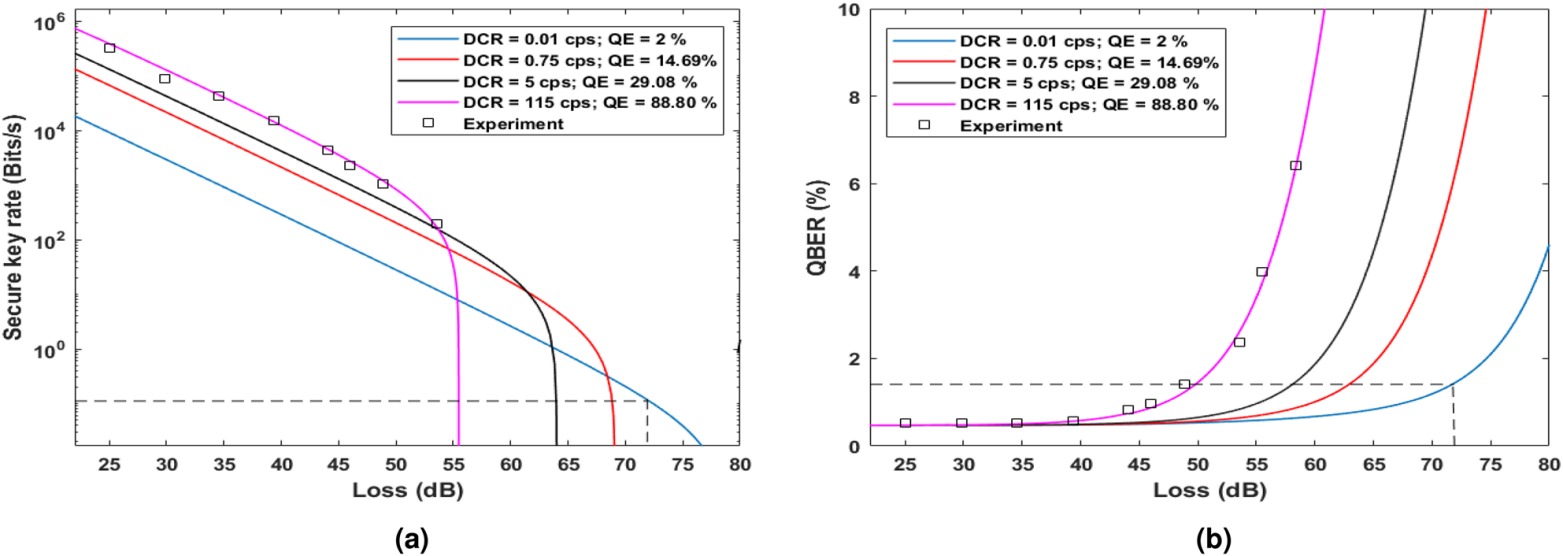
(**a**) Secure key rate and corresponding (**b**) QBER plotted with channel losses on variation in parameters of SNSPD. Decreasing the quantum efficiency (QE) of SNSPD also decreases the both dark count rate (DCR) and the secure key rate but increases the possible tolerable channel loss. A secure key rate of 0.11 bits/s is achievable at 72.2 dB loss (dash line) with QBER being only 1.48$\%$ by adjusting the dark count rate to 0.01 cps and quantum efficiency to 2$\%$.

One can see that in our study various sources of QBER are modeled and validated by experimental results. This theoretical analysis can be used in general for all phase-encoded QKD schemes such as phase-based BB84, RRDPS, and also for COW protocol. For a particular choice of laser in the experiment, the $QBER_{MZI}$ component is fixed, and the effect of chromatic dispersion could be ameliorated using a DCF, but the dark count rate of the single photon detector ultimately determines the maximum channel length possible to establish secure communication. We experimentally adjusted the quantum efficiencies of SNSPDs above 85$\%$ to obtain a high key rate. This experiment produces the highest key rate in DPS QKD protocol to date for all lengths. The optimization techniques described in this work helped us to suppress the QBER extraordinarily to 0.53$\%$ at 115 km and below 1$\%$ up to 225 km. From the $QBER_{disp}$ plot in Fig. 4a, it was observed that the $QBER_{disp}$ component grows fast at higher lengths. However, adding a DCF before the channel is a viable solution for managing dispersion for QKD using fibers. Furthermore, it does not add any additional loss to the system. A secure key generation through standard telecom fiber compatible with ITU-T recommendation G.652 was achieved. This enables the experimental setup for inter-city quantum communication for lengths more than 275 km with 0.19 dB/km fiber loss. This is the first implementation of DPS QKD for such lengths. This experiment also demonstrates the feasibility of DPS QKD using FPGA at 2.5 GHz without bulky hardware and using existing telecom infrastructure. The efficiency and dark count rates of SNSPDs depend on the detector bias currents similar to Fig. 2 in Ref.^51^. The detector bias current can be adjusted to reduce the dark count rate significantly at the expense of reducing the quantum efficiency. Figure 9a,b show the effect of lowering the dark count rate for our SNSPD on the system’s secure key rate and QBER, respectively. One can see that our optimized QKD setup can attain a 0.11 bits/s secure key rate with 72.2 dB channel loss with QBER of 1.48$\%$. In terms of length, the experimental setup is capable of producing 0.11 bits/s secure key rate at 380 km channel length. A comparison of available DPS QKD implementations in shown in Table 1. It is apparent that our present work demonstrates largest QKD channel length with least QBER value with 2.5 GHz clock rate. Furthermore in this experimental setup, by just replacing the current fiber with ultra-low loss (ULL) fiber having loss 0.15 dB per km, one can accomplish 0.11 bits/s secure key rate at 432 km channel length.

**Table 1:** Comparision of DPS QKD implementations.

Experiment	Clock rate	QBER	Secure KR	Channel length	Year
Wang et al.^28^	2 GHz	3.45$\%$	N.A.$^\text {a}$	260 km	2012
Diamanti et al.^31^	1 GHz	3.40$\%$	166 bps	100 km	2006
Takesue et al.^30^	10 GHz	> 4$\%$	12.1 bps	200 km	2007
Takesue et al.^30^	1 GHz	> 2.3$\%$	17 kbps	105 km	2007
Zhang et al.^42^	2 GHz	3$\%$	1.3 Mbps	10 km	2009
Shibata et al.$^\text {b}$^33^	1 GHz	2.93$\%$	0.03 bps	336 km	2014
This work$^\text {c}$	2.5 GHz	1.48$\%$ (2.36$\%$)	0.11 bps (192.7 bps)	380 km (265 km)	2023

$^{\text {a}}$ data not available in the literature. $^\text {b}$ This work used dispersion-shifted fiber as the quantum channel. $^\text {c}$ The values show the performance at minimum dark count settings of detectors. The values in the parenthesis correspond to highest efficiency of the detectors.

## Conclusion

In this work, we present a comprehensive model incorporating various sources of QBER in practical QKD systems similar to DPS QKD. We experimentally validate the outcomes of the theoretical model by performing one of the highest clock rate DPS QKD experiment so far (at 2.5 GHz) employing a portable FPGA for synchronization. Thanks to the optimized parameters given by the model, QKD for fiber length of 265 km is achieved with a QBER of $2.36\%$ and secure key rate of 193 bits/s. This successful demonstration paves a way for the straightforward implementation of the DPS QKD protocol with existing telecom infrastructure without specialty fibers and extending the scope for even higher clock rates in near future. The low QBER and its stability in this reported experiment makes the QKD link more tolerant against various environmental disturbances enabling for long time operation. The proposed theoretical model can be generalized to all those QKD protocol based practical systems that utilize coherent laser pulses, phase encoding, and Mach–Zehnder delay line interferometer leading to optimization of QKD systems using RRDPS, phase-based BB84, and COW protocol. This work also highlights that dispersion management using DCF is a compatible solution with the existing standard telecom system without needing dispersion-shifted fibers. By tuning the dark count rates of the detector, we show that the setup is capable of distributing secure keys for more than 380 km. This would significantly reduce the need for intermediate trusted nodes for such long distances, making the system more robust for ascertaining the security of QKD. This characterization method is a crucial step toward the commercial production of long distance secure practical QKD devices. This experiment motivates future developments enabling more than 432 km of secure key distribution with specialty low-loss fiber. With further development of high efficiency and low dark count single photon detectors, DPS QKD would be one of the top choices for metropolitan and intercity quantum networks.

## Data Availability

The data that support the findings of this study are available with the corresponding author upon reasonable request.
